# Recent evidence on quality of life following total gastrectomy for gastric cancer: A scoping review

**DOI:** 10.3892/mi.2026.314

**Published:** 2026-04-03

**Authors:** Malvina Eleftheriou, Dimitrios Ampazis, Abraham Pouliakis, Michael Doulberis, Konstantinos Toutouzas, Georgios Zografos, Dimitrios Theodorou, Tania Triantafyllou

**Affiliations:** 1 1st Department of Propaedeutic Surgery, Athens Medical School, National and Kapodistrian University of Athens, Hippokration General Hospital, 11527 Athens, Greece; 2 Department of Surgery, Royal College of Surgeons in Ireland, Connolly Hospital Blanchardstown, D15 K2DK Dublin, Ireland; 3 Respiratory Department, Cavan and Monaghan Hospital, HSE/RCSI Hospital Group, H12Y7W1 Cavan, Ireland; 4 Royal College of Surgeons in Ireland, University of Medicine and Health Sciences, D02 YN77 Dublin, Ireland; 5 2nd Department of Pathology, National and Kapodistrian University of Athens, ‘Attikon’ University Hospital, 12461 Athens, Greece; 6 Division of Gastroenterology and Hepatology, Department of Medicine, Zurich University Hospital, Zurich 8091, Switzerland; 7 Division of Gastroenterology and Hepatology, Medical University Department, Kantonsspital Aarau, Aarau 5001, Switzerland

**Keywords:** gastric cancer, gastrectomy, quality of life, patient-reported outcome measures, recovery of function

## Abstract

As survival rates improve following total gastrectomy for gastric cancer, quality of life (QoL) has become a critical assessment outcome alongside traditional oncological metrics. Both the short- and long-term course of recovery can be affected by post-gastrectomy syndromes and other post-operative outcomes; however, QoL remains underreported and underutilised in clinical evaluations. Herein, a scoping review of studies published after 2020 that assessed QoL following total gastrectomy for gastric cancer was performed, focusing on curative resections without additional major organ removal. Articles were identified through the PubMed, Scopus and EMBASE databases using combinations of key words related to gastrectomy, QoL and validated patient-reported outcome instruments. A total of nine studies met the inclusion criteria. The majority of functional outcomes (physical, role and emotional functioning) consistently declined at an early stage following the surgery, with recovery typically beginning after ~6 months. Key symptoms, particularly reflux and eating restrictions, remained variable and could persist for up to 2 years. Global QoL improved over time despite ongoing symptoms, suggesting a degree of psychosocial adjustment. Surgical approach and anastomotic technique had limited long-term impact, though minimally invasive methods have been reported to show modest short-term advantages. Despite its clinical importance, QoL remains an underused primary outcome in gastric cancer surgery. A stronger focus on standardised, patient-centred assessment could bridge the gap between technical success and meaningful recovery. Identifying the distinct course of QoL following total gastrectomy may facilitate its integration into surgical planning and care. To the best of our knowledge, this is the first scoping review to focus exclusively on QoL following total gastrectomy in the era of updated surgical guidelines, providing an up-to-date framework for improving patient outcomes.

## Introduction

Gastric cancer (GC) ranks as the fifth most prevalent malignancy worldwide and is also the fifth leading cause of cancer-related mortality (1). For patients with resectable disease, total gastrectomy with D2 lymphadenectomy remains the standard curative surgical approach, typically followed by gastrointestinal reconstruction (2,3). Depending on pre-operative staging and risk assessment, certain patients may receive neoadjuvant therapy, while adjuvant chemotherapy or chemoradiotherapy may be indicated based on pathological findings, in accordance with widely accepted international guidelines (2-4). According to the 8th edition of the Tumour Node Metastasis (TNM) classification, the 5-year survival rates following R0 resection have improved significantly compared to previous decades, ranging from 89.9% in stage IB cases to 20.2% in stage IIIC cases (5).

As survival outcomes have improved, health-related quality of life (QoL) has gained increasing attention in GC care, a domain historically underemphasized in surgical and oncological literature (6-8). Total gastrectomy, despite its curative intent, often leads to persistent post-gastrectomy syndromes, such as nutritional deficiencies, digestive dysfunction and psychosocial challenges, all of which can significantly impair long-term QoL (8-11). Recognising this, there is a growing emphasis on integrating QoL as a core outcome measure in both clinical trials and routine care (9,11,12). QoL may be significantly impaired even in patients with otherwise favourable clinical outcomes. Notably, QoL has been identified as an independent prognostic factor for survival, underscoring its importance in the post-operative management of patients undergoing total gastrectomy (6,12).

Given its growing clinical relevance, it is important to understand what QoL truly encompasses. QoL is a multidimensional concept that includes physical, psychological, social, and functional well-being, shaped by individual goals, cultural context, and expectations. This holistic perspective reinforces the need to balance oncological success with the overall lived experience and recovery experienced by the patient (11,12).

To measure QoL in a meaningful manner, patient-reported outcome (PRO) assessments are most commonly used, which capture the experiences of patients directly, without clinician interpretation. PRO assessments provide insight beyond clinical metrics, assisting in the evaluation of the real-world impact of surgery, as well as in both treatment decisions and long-term care strategies (6,13).

Questionnaires are essential tools for documenting the experience of a patient and assessing QoL. The most widely used instrument for patients with cancer is the European Organization for Research and Treatment of Cancer Quality of Life Questionnaire-Core 30 (EORTC QLQ-C30; https://qol.eortc.org/questionnaires/) (14), a 30-item tool that evaluates multiple dimensions of QoL. It includes five functional scales (physical, role, cognitive, emotional and social functioning), a global QoL scale, three symptom scales (fatigue, nausea/vomiting and pain), and six single-item measures addressing appetite loss, diarrhoea, dyspnoea, constipation, insomnia and financial difficulties (6,9).

To complement the generic QLQ-C30, the EORTC QLQ-STO22 (https://qol.eortc.org/questionnaire/gastric-cancer-update-of-qlq-sto22/) module was developed specifically for patients with GC (15). It consists of 22 items assessing disease-specific issues such as dysphagia, early satiety, reflux, taste disturbances, eating-related anxiety, pain and body image concerns. Collectively, the QLQ-C30 and QLQ-STO22 provide both a general and a disease-specific evaluation of the QoL of patients with GC.

The Postgastrectomy Syndrome Assessment Scale (PGSAS) is a disease-specific tool developed to evaluate QoL after gastrectomy. The PGSAS-37, derived from the original 45-item version, is organised into three domains: Symptoms (oesophageal reflux, abdominal pain, meal-related distress, indigestion, diarrhoea, constipation, dumping) plus a total symptom score; living status measures (food intake, need for additional meals, meal quality and ability to work); and QoL subscales on dissatisfaction with symptoms, meals, work and daily life (16,17).

The EuroQol 5 Dimensions (EQ-5D) is a generic, standardised instrument developed by the EuroQol Group to assess QoL across five dimensions: mobility, self-care, usual activities, pain/discomfort and anxiety/depression. It also includes a visual analogue scale for rating overall health (18).

Several other instruments have been created to assess the QoL of patients with GC, including the Short Form-36 Health Survey (SF-36) (19), the Functional Assessment of Cancer Therapy-Gastric (FACT-Ga) (11), the MD Anderson Symptom Inventory-Gastrointestinal Module (MDASI-GI) (13) and the Korean Quality of Life in Stomach Cancer Surgery (KOQUSS) (20) questionnaire. While some of these tools assess overlapping domains, others, such as the KOQUSS, were specifically designed to capture post-gastrectomy experiences. Despite validation in selected languages, the broader international use of these instruments remains limited.

It is evident that certain domains, such as physical functioning, pain and social functioning, are consistently represented across different questionnaires, allowing comparison, whereas others are unique to individual tools, capturing aspects that may not be addressed elsewhere; recognising these overlaps and distinctions is essential for meaningful interpretation and for structuring the results in a manner that facilitates cross-study and cross-instrument analysis.

Moreover, the interpretation of these questionnaires, although described in detail within the manual of each instrument, varies between tools. Each questionnaire uses a different scoring approach; for example, the PGSAS is based on raw scores, whereas the EORTC can be calculated from either raw scores or linear transformations, with a distinct transformation algorithm for each domain (9). Consequently, this necessitates separate analyses for each domain. It should be noted that higher scores do not always indicate a better QoL; for example, in symptom scales, a higher score reflects greater symptom burden and therefore poorer QoL (10,16,17,21). Certain studies have explored the use of an overall ‘summative score’ (6,22); however, the majority of researchers analyse outcomes on a domain-by-domain basis (8,9,12,15).

The aim of the present scoping review was to examine recent evidence on post-operative QoL following total gastrectomy, with a focus on both its chronological course and variations related to surgical technique. Studies published within the previous 5 years were used to reflect research conducted after major updates to GC treatment guidelines, introduced in the West in 2013(23) and in the East in 2016(24). These updates redefined the standard surgical approach by excluding routine removal of spleen and pancreas from curative total gastrectomy. Given the significance of this change, it is reasonable to expect that it has had a substantial impact on the QoL of patients with GC, making it a relevant factor to consider in recent studies. To the best of our knowledge, this is the first scoping review to specifically examine QoL following total gastrectomy in the context of the most recent major guideline updates, providing a timely perspective on outcomes in the modern surgical era.

## Data and methods

A comprehensive search of the PubMed, Scopus and EMBASE databases was performed using combinations of the terms\total gastrectomy\,\gastric cancer\,\quality of life\,\patient-reported outcomes', as well as the names of commonly used QoL instruments (e.g., EORTC QLQ-C30, QLQ-STO22, PGSAS-37 and EQ-5D). These terms were combined using Boolean operators (AND/OR) as appropriate. To capture the most recent data, the search was limited to studies published between 2020 and 2025, with the aim of including research conducted after the most recent major updates in GC treatment guidelines. The search and initial screening were performed by the first author, and the selection of eligible studies was discussed with the co-authors. Titles and abstracts were initially screened for eligibility, followed by full-text assessments of potentially relevant studies. Any discrepancies were resolved through discussion and consensus among the authors.

The present scoping review was conducted and reported in accordance with the Preferred Reporting Items for Systematic Reviews and Meta-Analyses extension for Scoping Reviews (PRISMA-ScR) guidelines (25).

Studies were eligible if they investigated total gastrectomy, reported QoL outcomes using validated questionnaires and explicitly referred to curative total gastrectomy without any additional major organ resection. Non-English-language studies, those conducted for non-oncological indications, those lacking explicit QoL outcomes, or those not exclusively focused on total gastrectomy were excluded. The search was limited to studies published from 2020 onwards to reflect contemporary clinical practice following the widespread adoption of recent guideline updates. Studies involving multivisceral resections or comparing total to subtotal gastrectomy without extractable data specific to total gastrectomy were also excluded.

**Figure 1:**
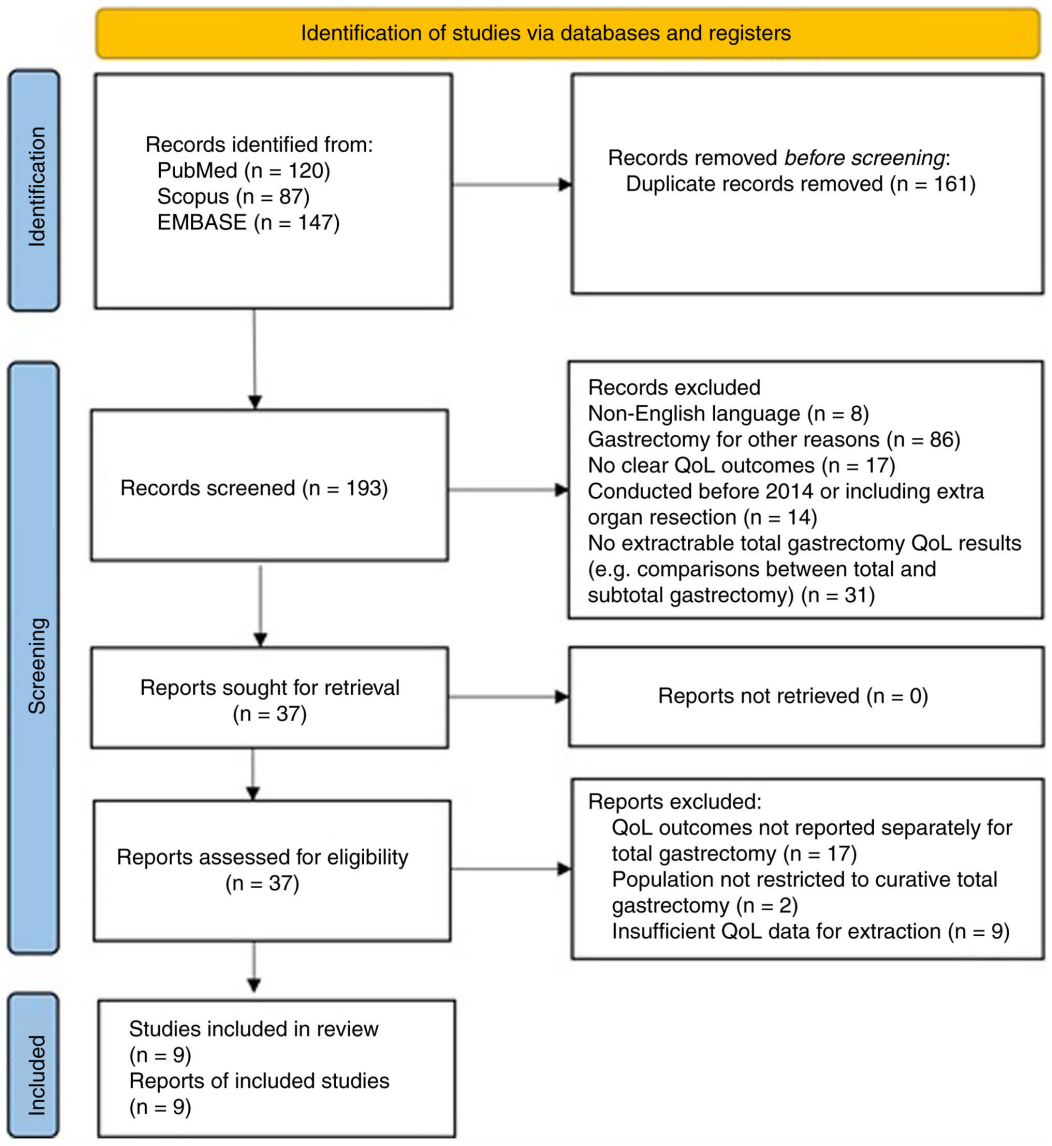
PRISMA-ScR flow diagram of the study selection process. Flowchart illustrating the identification, screening, eligibility assessment, and inclusion of studies evaluating quality of life after total gastrectomy. The diagram summarizes the number of records identified through database searching, duplicates removed, records screened, full-text articles assessed for eligibility, and the final studies included in this scoping review. PRISMA-ScR, Preferred Reporting Items for Systematic Reviews and Meta-Analyses extension for Scoping Reviews.

Following the removal of duplicates and applying the predefined inclusion and exclusion criteria, a total of nine studies were selected for analysis, as outlined in the PRISMA-ScR flowchart (Fig. 1).

## Results

A total of nine studies met the predefined eligibility criteria and were included in the final analysis (Fig. 1). Among these, Wei *et al* (26) and Yan *et al* (27) conducted retrospective analyses comparing, among other outcomes, QoL following linear stapling (LS) vs. circular stapling (CS) for the esophagojejunal anastomosis. Wei *et al* (26) supplemented their assessment with the Gastroesophageal Reflux Disease Questionnaire (GERDQ) in order to more accurately capture reflux symptoms. In addition, Yan *et al* (27) compared intracorporeal vs. extracorporeal anastomosis, along with various anastomotic configurations, such as overlap and π-shaped, as well as the OrVil-assisted technique. Lin *et al* (28) compared QoL outcomes between totally laparoscopic total gastrectomy (TLTG) and laparoscopic-assisted total gastrectomy (LATG). Van der Wielen *et al* (29) assessed QoL outcomes between open total gastrectomy (OTG) and minimally invasive total gastrectomy (MITG) in patients with advanced GC treated with neoadjuvant chemotherapy. A similar prospective comparison between open and laparoscopic total gastrectomy was conducted by Tanaka *et al* (17). Saeki *et al* (21) correlated high-resolution manometry (HRM) findings with QoL, while Lu *et al* (30) retrospectively compared QoL outcomes between patients undergoing standard Roux-en-Y anastomosis (RY) and those receiving proximal jejunal pouch Roux-en-Y anastomosis (PP-RY). Kubota *et al* (16) compared QoL between elderly and non-elderly patients undergoing aboral pouch reconstruction. Finally, Park *et al* (9) conducted a prospective longitudinal study over a period of 3 years comparing QoL after total and distal gastrectomy. Although the latter study would normally be ineligible, it was the only study under consideration to present clear, separate data on chronological changes in QoL domains for the total gastrectomy subgroup. These data, which were explicitly reported and independent from the study's other outcomes, were used as a foundation for the assessment of how QoL evolved after total gastrectomy. An outline of the characteristics of the included studies is presented in Table I.

The majority of the included studies were conducted in East Asia, with the majority designed retrospectively. The timepoints for questionnaire distribution ranged from the pre-operative period up to 3 years post-operatively. In total, 990 cases were analysed in the studies. Given the exploratory nature of the present scoping review, the aim was to synthesise recent evidence and identify patterns in QoL outcomes rather than to perform a formal methodological appraisal of individual studies.

The eligible studies included in the present scoping review used various QoL instruments: EORTC QLQ-C30, QLQ-STO22, EQ-5D and PGSAS-37. Building on the observed overlap between certain domains, outcomes were organised into conceptually grouped categories to facilitate consistent comparison across instruments, with the EORTC framework used as the primary reference given its widespread use and established structure. The domains and corresponding questions from other instruments were then mapped and adapted accordingly, allowing for alignment under common thematic categories (Table II). To the best of our knowledge, this approach has not been previously described and aims to improve clarity and comparability across studies. The findings are therefore presented according to the unified domains. For each domain, a definition is provided, followed by an overview of its chronological evolution and a comparison across surgical techniques.

### Functioning scales

The functioning scales are demonstrated in Tables III and IV.

*Physical performance and functional capacity*. This domain encompasses the recovery of mobility, self-care and general physical strength following total gastrectomy.

In terms of chronological patterns, an early decline with recovery complete by 6-12 months was found to be consistent across the cohorts. At >12 months, recovery remained below the baseline in adjuvant-exposed minimally invasive surgery (MIS) cohorts (9,28) whereas early-stage (17), neoadjuvant-managed mixed-stage (29) and mixed-stage MIS cohorts without reported chemotherapy (26) returned to baseline. Comparing the various techniques, physical performance outcomes were shown to be comparable, with no significant differences reported between stapling methods, surgical approaches or reconstruction types (16,17,26-30).

*Role engagement and daily living.* ‘Role functioning’ reflects the ability of the patient to resume work, leisure activities and daily responsibilities following total gastrectomy.

In early-stage (17) and neoadjuvant-managed mixed-stage (29) cohorts, role functioning was found to decline initially, but returned to the baseline within the first year. By contrast, in mixed-stage MIS cohorts with adjuvant therapy (28), improvements were observed, although baseline levels were not restored, whereas advanced-stage patients exposed to adjuvant therapy showed a sustained decline (9).

Comparative analyses subsequently revealed limited technique-associated differences in role functioning. In mixed-stage MIS cohorts without neoadjuvant therapy, return to work improved following TLTG compared with LATG, although only from 6-12 months onward (28). Open cohorts reported worse role functioning compared with MIS cohorts, although these differences were found not to be significant (29).

*Social integration and interaction.* ‘Social functioning’ concerns how effectively a patient can engage in social activities, maintain interpersonal relationships and adapt to post-gastrectomy lifestyle changes.

Chronological trajectories were found to vary among the cohorts. In advanced-stage MIS patients with adjuvant therapy, the scores improved initially, peaked at 1 year, and subsequently declined (9). In mixed-stage groups, the OTG cohort returned to baseline by 12 months, and later exceeded it, whereas the MITG cohort stayed below the baseline throughout follow-up (29).

When the techniques were compared, in some studies, open surgery cohorts scored higher than the MIS cohorts; the OTG group returned to baseline by 6 months, whereas the MITG group declined from 3 months onward (17,29). The patients who underwent TLTG exhibited improved social interaction compared with patients who underwent LATG during the first post-operative year, although this improvement was found not to be statistically significant (28). Increased meal frequency was also reported in early-stage cohorts, potentially affecting daily living patterns (21).

*Emotional and psychological well-being.* This domain captures the patients' emotional functioning and psychological distress, primarily assessed using the EORTC QLQ-C30 questionnaire and STO22 module (anxiety domain), with additional contribution from the EQ-5D instrument (anxiety/depression dimension).

Emotional outcomes varied according to the cohort and over time. Advanced-stage patients with MIS who received adjuvant therapy showed early improvement that declined after 1-2 years (9), whereas early-stage patients treated without systemic therapy experienced only a transient early decline, followed by recovery (17). Mixed-stage MIS cohorts generally reported stable scores, with occasional transient improvement that returned to baseline (26-30).

Technique-associated effects on emotional functioning were found to be limited. PP-RY was associated with improved anxiety scores compared with standard RY (30). Stapling methods exhibited no consistent impact, although patients who underwent CS tended to report more anxiety at 12 months compared with those who underwent LS (26). MIS cohorts occasionally demonstrated slightly improved emotional scores at 6 months, although the overall differences compared with open surgery patients were found not to be not significant; open cohorts occasionally reported higher body image scores, although again, these were not statistically significant (27-29). Furthermore, pouch reconstructions exhibited no significant differences in either emotional or body image outcomes (16,30).

Body image was found to be comparable across reconstructions and technical variations, including pouch type, stapling method, age-related pouch use and the surgical approach (16,21,30).

*Cognitive resilience.* ‘Cognitive resilience’ relates to the ability of a patient to maintain concentration, memory and mental clarity during recovery. It is specifically assessed in the EORTC QLQ-C30 questionnaire, although it is not routinely captured by the majority of other QoL instruments used in post-gastrectomy studies.

Over time, a decline was observed during the first year with only minimal recovery in advanced-stage MIS cohorts who were receiving adjuvant therapy (9), whereas early-stage and mixed-stage cohorts managed largely without systemic therapy maintained stable scores throughout follow-up (17,26,27,29,30).

Finally, no significant differences in cognitive outcomes were reported across stapling methods, surgical approaches or pouch reconstructions (17,27,28,30).

### Single items. Global health perception and life satisfaction

This domain represents the subjective well-being, satisfaction or dissatisfaction and adaptation to life of a patients following total gastrectomy.

The course of global health recovery differed among the cohorts. In mixed-stage groups with neoadjuvant therapy, the scores declined early on, improved by 1 year, and returned to the baseline only with the OTG group, whereas the MITG group remained below baseline throughout follow-up (29). By contrast, advanced-stage MIS cohorts with adjuvant therapy exhibited gradual improvement over time (9).

In terms of comparing techniques, TLTG was associated with earlier improvements compared with LATG in a mixed-stage MIS cohort who did not receive neoadjuvant therapy (28). MIS was associated with higher global health scores in some cohorts compared with open surgery in mixed-stage groups who were treated with neoadjuvant therapy (29). Finally, PP-RY reconstructions were found to be associated with an improved overall quality of life compared with standard RY (30).

With regard to dissatisfaction, no significant differences were observed across the stapling techniques, between elderly and non-elderly patients, or between MIS and open surgery approaches (16,17,21).

*Economic consequences.* Financial difficulties were assessed in a subset of studies as a measure of post-operative socioeconomic burden. This domain was not evaluated in studies utilizing the PGSAS instrument.

Chronological patterns were found to be largely stable across the studies. The majority of the cohorts, including early- and mixed-stage groups managed with MIS or open approaches, reported no significant changes over time (17,26-30). By contrast, patients with advanced-stage MIS exposed to adjuvant therapy experienced an increase in financial strain during the second year, with recovery by the 3rd year (9).

Upon comparing the techniques, the stapling method appeared to influence financial outcomes, with LS associated with improved scores at 12 months compared with CS in mixed-stage MIS cohorts (26,27). Open surgery groups reported higher burden scores than the MIS groups in early-stage cohorts, although these differences were found not to be statistically significant (17).

### Symptom experience and disease burden

A detailed summary of the chronological evolution of symptom-related QoL outcomes following gastrectomy is provided in Table V. The most concerning post-gastrectomy symptoms, indicatively the eating restrictions, pain, diarrhoea and reflux, worsened in the early post-operative period, and gradually improved within the first year, although reflux often persisted, whereas eating restrictions were slower to improve and sometimes remained in the long term.

Variations across studies were observed in the context of differing patient characteristics and treatment exposures. Prolonged fatigue, pain, diarrhoea and taste disruptions were found to be more pronounced in advanced-stage patients, the majority of whom received adjuvant therapy (9), whereas earlier recovery was described in mixed-stage cohorts treated with neoadjuvant therapy (29). Stable or improving outcomes were observed both in early-stage cohorts (17) and in mixed-stage MIS groups where chemotherapy exposure was absent or not reported (26-28).

Differences in recovery trajectories were also observed between surgical techniques. Notably, minimally invasive approaches were reported in some studies to be associated with slower resolution of pain compared with open surgery (9,29). By contrast, in a single study, totally laparoscopic procedures were found to be associated with an earlier improvement in reflux compared with laparoscopic-assisted techniques (29), although this observation was made from a single study, and therefore should be interpreted with caution.

Table VI summarises comparative findings in symptom-associated QoL. Symptom outcomes were similar across studies regardless of stage distribution or perioperative therapy. Most of the technique-associated comparisons revealed no significant differences; when present, the differences were small and inconsistent. Within MIS, the stapler type and anastomotic method were associated with isolated differences, including less constipation and dysphagia, but more cases of early diarrhoea and worse reflux in certain subgroups (26,27). A short-term advantage was observed for TLTG over LATG at 6 months, although without long-term differences (28). The reconstruction method also appeared relevant, with PP-RY associated with reduced pain and improved appetite outcomes compared with standard RY (30).

### Influence of other clinical variables

In addition to questionnaire domains, the present scoping review recorded whether the studies reported on general health status, peri-operative therapy or post-operative complications, as these may influence QoL outcomes. Adjuvant therapy was described in two studies (9,28), whereas three reported on neoadjuvant therapy (27-29). Post-operative complications were mentioned in five studies (16,17,27,28,30). However, none of the studies analysed these variables in conjunction with QoL.

## Discussion

QoL is increasingly recognised as a key outcome in cancer care, reflecting not only treatment success but also the ability of a patient to adapt and recover following major interventions, such as total gastrectomy. While oncological results remain essential, understanding functional recovery is equally important and may guide surgical decision-making (26).

The present review found that physical, role and emotional functioning consistently declined at an early stage post-operatively, with recovery typically beginning ~6 months. The pattern observed in the present scoping review is in agreement with other mixed gastrectomy studies (12,15). While this early decline is partly expected as the body heals from a major surgery, it is also influenced by post-gastrectomy symptoms that can significantly affect daily functioning (12). Moreover, this period often coincides with the initiation of adjuvant chemotherapy or chemoradiotherapy for several patients, which can further impact QoL. While recovery to baseline values may occur by 6 months, it should be noted that baseline does not necessarily reflect an optimal state, particularly in patients with advanced disease (9,29). As highlighted in the wider literature, recognising this trajectory can support patient expectation management and inform tailored rehabilitation planning (8,9).

The present scoping review identified mixed outcomes on social functioning following total gastrectomy. The studies demonstrating an early decline are in agreement with existing literature; Hu *et al* (15) reported a marked decline in social functioning after surgery, particularly during the first 45 post-operative days, with subsequent improvement to near-baseline levels. Vaccaro *et al* (31) observed a similar pattern, suggesting that changes in body image, disruption of established routines and pleasures, and the need to adapt to new eating patterns may hinder social engagement, particularly during meals. While a decline in social functioning over time is well documented in the literature, the improvement reported in some studies, particularly at ~12-months, may reflect the gradual adaptation of patients to post-operative changes and the resumption of social activities (17,29). Surgical approach did not appear to have a consistent or statistically significant impact on social functioning. Given the multifactorial nature of this domain, including physical recovery, nutritional adaptation, emotional well-being, and social support, isolating the effect of surgery alone is challenging. Longitudinal, standardised assessment may be needed to clarify the true trajectory of social functioning after total gastrectomy.

In the present scoping review, only the studies that used the EORTC QLQ-C30 questionnaire assessed cognitive status. Of note, one study reported a measurable decline (9), while the others found no change over time or variation based on surgical technique (17,26,27,29,30). Overall, the available evidence is insufficient to draw conclusive interpretations in this domain, and several factors may account for this inconsistency. A likely contributing factor is the early timing of assessment in certain studies. This may have captured short-term post-operative cognitive dysfunction, which typically resolves within 3 months (32,33). Another contributing factor may be the limited structure of the cognitive domain within these QoL instruments, which typically includes only two questions and does not constitute a formal cognitive assessment, such as the Mini-Mental State Examination (34). As such, their sensitivity to subtle or transient cognitive changes may be limited. The aforementioned considerations highlight the need for more thoughtful application and interpretation of existing QoL tools when assessing post-operative cognitive function.

The present scoping review found no evidence of significant financial strain over time between subgroups; a non-significant trend favouring linear over circular stapler techniques may relate to the higher post-operative complication rates reported with circular stapling, including bleeding, anastomotic stenosis and dysphagia (26,27). The only study demonstrating a decrease in financial burden involved early-stage GC, suggesting the improvement may be linked to a reduced need for post-operative therapy rather than surgical approach (17). Literature on oncologic surgery for upper gastrointestinal cancers, highlights that chemotherapy, financial demands of treatment-including personal expenses, travel and accommodation for care- and other related costs, can place a considerable strain on patients' economic well-being (35,36). Given the distinct patient groups and the varied socioeconomic contexts across study sites, firm conclusions regarding the financial domain remain challenging.

Symptom trajectories appeared to vary according to patient and treatment characteristics rather than surgical approach alone. Patients with advanced-stage disease receiving adjuvant therapy experienced more prolonged fatigue, pain, diarrhoea and taste disruptions (9), whereas mixed-stage cohorts treated with neoadjuvant therapy reported earlier recovery in several domains (29). Stable or improving symptom profiles were more often observed in early-stage cohorts (17) and in mixed-stage MIS groups without chemotherapy exposure (26-28). These findings suggest that disease stage and peri-operative therapy may exert greater influence on symptom burden than surgical technique itself. Variability in follow-up schedules and symptom reporting tools further complicates interpretation, underscoring the need for longitudinal, standardised assessment to clarify how patient and treatment factors interact in shaping post-operative symptom profiles.

The observation that global QoL improved even when symptoms persisted suggests that overall well-being is not simply the cumulative effect of individual complaints, but also reflects the capacity of patients to adapt and reframe their post-gastrectomy experience. Similar findings have been described in other oncologic populations, where coping mechanisms, resilience, and social support play a decisive role in maintaining global QoL despite ongoing treatment-related burdens (35,36). This perspective highlights the importance of integrating psychosocial and rehabilitative support into survivorship care, in parallel with symptom management. While surgical modifications such as minimally invasive or pouch reconstructions may influence short-term recovery, their impact on long-term global QoL appears limited when compared with the broader determinants of adaptation and support.

Overall, the patterns observed across the included studies suggest that post-operative QoL trajectories following total gastrectomy may be influenced more strongly by disease stage and exposure to systemic therapy than by differences in surgical technique alone. Nevertheless, interpretation of the patterns observed across the reviewed studies should be undertaken with caution, as the included studies differ substantially in stage distribution, exposure to perioperative systemic therapy, and timing of QoL assessment. It would indeed be of considerable interest to better understand the independent contribution of surgical technique, systemic therapy, and disease-related factors to post-operative QoL, particularly the effect of each factor considered in isolation. In the currently available literature, however, these elements are closely intertwined within diverse patient populations, and the combination of sample size and study design limits the ability to isolate their individual effects. Further studies specifically designed to address these questions may help clarify the relative contribution of each factor.

Reviewing the current literature revealed several key concerns regarding QoL assessment following total gastrectomy. While QoL is increasingly recognised as an important component of GC care, relatively few studies consider it as a primary outcome, with the majority still focusing on traditional surgical endpoints such as complications, resection margins, and survival. When QoL is assessed, it is often used to compare surgical techniques or patient subgroups rather than to chart the full course of recovery. This tendency provides only a partial view of recovery, limiting insight into the longer-term physical, emotional and social challenges faced by patients after surgery.

Another dimension of heterogeneity relates to geography. The present scoping review found a marked imbalance in QoL research on curative total gastrectomy over the past 5 years, with the majority of studies conducted in East Asia, where GC is more common (1). While these data provide valuable clinical insight, the predominance of Asian study populations limits the generalisability of findings, particularly in culturally sensitive domains such as role functioning, dietary practices and social engagement. Notably, despite the development of one of the most comprehensive and widely validated QoL instruments for cancer patients, the EORTC QLQ-C30 and its gastric-specific module, Western centres have contributed relatively few QoL studies focused specifically on total gastrectomy. Addressing the regional imbalance requires consideration of cultural and geographical context when interpreting QoL instruments and when formulating clinical recommendations.

Building on the challenges outlined above, a major difficulty in interpreting QoL outcomes is the marked heterogeneity among available studies. This includes differences in study design, such as retrospective and prospective approaches, differences in setting between multicentre and single-centre studies and variations in focus, with certain studies comparing surgical techniques within specific subgroups. Such variability limits the generalisability of findings and complicates efforts to compare and synthesise results across the literature.

Marked variability in the timing of QoL distribution further complicates interpretation. Only a small number of studies applied questionnaires pre-operatively or in the early post-operative period (9,17,27,29), when physical and psychological effects are most acute. While certain studies tracked changes over time (9,21,17,28), others relied on a single time point (16,26,27,29,30). The fact that QoL is primarily considered in relation to long-term outcomes may explain why questionnaires are not commonly used to capture the impact of short-term complications, which are often described under the separate concept of ‘quality of recovery’ (37,38). Yet, nothing in the instrument manuals precludes their early use, leaving an opportunity to better understand the immediate post-operative experience; an approach that could yield valuable insights into the trajectory from short-term recovery to long-term well-being (12,27).

These methodological differences are further influenced by the limitations of the instruments themselves. In the present scoping review, no single questionnaire was found to be capable of comprehensively assessing QoL following total gastrectomy. The EORTC QLQ-C30, developed for patients with cancer, covers a broad range of domains, but requires the STO22 module to capture GC-specific symptoms, resulting in 52 questions, which can be burdensome for patients and may reduce response rates. Similarly, researchers using the PGSAS-37 often supplemented it with the EORTC or omitted domains, such as functioning and financial status entirely. Overlap between certain domains and omission of others not only limits comparability between studies, but also impedes the ability to track the full course of recovery. The lack of uniformity in scoring and scale direction across instruments requires familiarity with each tool's methodology, increases the complexity of analysis, and makes cross-study comparisons more difficult. The aforementioned challenges emphasise the importance of applying existing instruments within a standardised framework to minimise respondent burden while ensuring all relevant domains are assessed.

Finally, although the majority of studies reported clinical variables, such as pre-operative treatment or post-operative complications, their potential influence on QoL outcomes was rarely explored. Most importantly, none of the studies considered the impact of post-operative chemotherapy on QoL, despite its recognised effect on recovery and overall well-being. These gaps are particularly critical, given that several patients with GC may already be malnourished, anxious, or psychologically burdened at the time of diagnosis. Without consideration of these variables, QoL results may reflect not only the effects of surgery but also broader clinical and psychosocial conditions. This lack of adjustment can confound observed QoL patterns, making it difficult to separate the effects of surgery from those of the underlying disease, treatment-related side effects, or pre-existing patient conditions. To improve interpretability and comparability, future research would benefit from clearly defined assessment timelines and a systematic evaluation of pre-operative status, post-operative course, and adjuvant therapies.

The present scoping review has certain limitations. As a scoping rather than a systematic review, it may not capture all available literature despite a focused and thorough search. Additionally, by limiting inclusion to patients undergoing total gastrectomy without additional major organ resection, certain relevant data may have been excluded, potentially affecting the completeness of the findings. Finally, the inconsistency in how QoL is assessed and reported across studies makes comparison challenging and may weaken the overall interpretability of results.

In conclusion, total gastrectomy remains a life-altering procedure with long-term consequences for the daily life of a patient. Beyond survival and surgical endpoints, QoL is a critical outcome as it can influence treatment decisions and guide both clinicians and patients through post-operative challenges. A clear understanding of QoL in both the early and late phases of recovery is essential for informing expectations and optimising follow-up strategies. Equally, evaluating how different surgical techniques shape these outcomes can provide valuable guidance for shared decision-making. To achieve this, more QoL-focused studies are needed, designed to capture both chronological changes and procedure-specific outcomes across a range of cultural and clinical settings.

## Figures and Tables

**Table I:** Characteristics of the studies included in the scoping review of QoL following total gastrectomy.

Authors, year of publication	Study type	Study period	Study geographical region	No. of cases	Total gastrectomy cases only	Questionnaire administration timeline	Comparison parameters	Questionnaires used	(Refs.)
Wei *et al*, 2021	Retrospective	2014-2018	China	120	Yes	Post-operatively after 6 months and 1 year	Linear stapler vs. circular Stapler	EORTC QLQ C-30 EORTC STO-22 GERDQ	(26)
Yan *et al*, 2023	Retrospective	2015-2020	China and USA	105	Yes	Pre-operatively and post-operatively after 1 year	i) Linear stapler vs. circular stapler; ii) Intracorporeal vs. extracorporeal anastomosis; iii) Overlap vs. π-shaped vs. OrVil anastomosis	EORTC QLQ C-30 EORTC STO-22	(27)
Lin *et al*, 2023	Retrospective	2014-2018	China	312	Yes	Post-operatively after 3 months, 6 months, and 1 year	TLTG vs. LATG	EORTC QLQ C-30 EORTC STO-22	(28)
Van der Wielen *et al*, 2022	Prospective-RCT	2015-2018	Europe	96	Yes	Pre-operatively and post-operatively after 5 days, 3 months, 6 months, and 1 year	Open vs. MIS gastrectomy in neoadjuvant patients	EORTC QLQ C-30 EORTC STO-22 EQ5D	(29)
Tanaka *et al*, 2024	Prospective	2015-2020	Japan	59	Yes	Pre-operatively and post-operatively after 1 month, 3 months, 1 year, and 3 years	Laparoscopic vs. open gastrectomy	EORTCC30 PGSAS 37	(17)
Saeki *et al*, 2024	Retrospective	2014-2020	Japan	12	Yes	Post-operatively after 1 year	i) HRM findings; ii) Linear stapler vs. circular stapler	PGSAS-37	(21)
Lu *et al*, 2022	Retrospective	2019-2020	China	136	Yes	Post-operatively after 1 year	RY vs. PP-RY	EORTC QLQ C-30 EORTC STO-22	(30)
Kubota *et al*, 2024	Retrospective	2016-2022	Japan	36	Yes	Post-operatively after 1 year	Aboral pouch in the elderly vs.non-elderly population	PGSAS-37	(16)
Park *et al*, 2020	Prospective	2011-2014	Korea	114	No	Pre-operatively, and post-operatively after 1, 2 and 3 years	TG vs. DG (TG data used only)	EORTC QLQ C-30 EORTC STO-22	(9)

DG, distal gastrectomy; EQ5D, EuroQol-5 Dimensions; EORTC, European Organisation for Research and Treatment of Cancer; HRM, high-resolution manometry; LATG, laparoscopically assisted total gastrectomy; MIS, minimally invasive surgery; OrVil, Oral-Villanueva stapler system; PGSAS 37, Postgastrectomy Syndrome Assessment Scale (37-item version); PGSAS-45, Postgastrectomy Syndrome Assessment Scale (45-item version); PP-RY, Proximal Jejunum Pouch Roux-en-Y; RCT, randomised controlled trial; RY, Roux-en-Y reconstruction; TG, total gastrectomy; TLTG, totally laparoscopic total gastrectomy.

**Table II:** Unified domains of QoL questionnaires following total gastrectomy.

Domain	EORTC QLQ-C30^a^	EORTC STO22	EQD5	PGSAS-37^b^
Functioning scales	Physical function	-	Mobility, self-care	-
	Role function	-	Usual activities	-
	Emotional function	Anxiety/body image	Anxiety/depression	-
	Cognitive function	-	-	-
	Social function	Trouble eating with others (item 46)	-	Meals (living status)
Symptom scales	Fatigue	-	-	-
	Nausea and vomiting	-	-	-
	Pain	Abdominal pain	Pain/discomfort	Abdominal pain
	Dyspnoea	-	-	-
	Insomnia	-	-	-
	Constipation/diarrhoea	-	-	Constipation/diarrhoea
	-	Reflux	-	Oesophageal reflux/indigestion
	-	Dysphagia	-	-
	-	Eating restrictions	-	Meal-related distress/Meals (ingestion)
	-	Taste	-	-
	-	Dry mouth	-	-
	-	Hair loss	-	-
	-	-	-	Dumping subscale
Single items	Global health/QoL	-	-	Dissatisfaction
	Financial impact	-	-	-

^a^Domains from the EORTC QLQ-C30 were used as the reference framework, and corresponding scales or items from the EORTC QLQ-STO22, EQ-5D, and PGSAS-37 were aligned accordingly.

^b^For the PGSAS-37, questions relating to meals were split; those referring to food ingestion were grouped under ‘eating restrictions’, while those reflecting the social context of meals were placed under ‘social functioning’. As shown, several domains overlap across instruments, while others are not represented, reflecting variations in the aspects of QoL that each tool assesses. QoL, quality of life; EORTC, European Organisation for Research and Treatment of Cancer Quality of Life Questionnaire; EQ-5D, EuroQol-5; PGSAS-37, Postgastrectomy Syndrome Assessment Scale-37.

**Table III:** Chronological patterns of functional and single-item quality of life following gastrectomy.

Authors, year of publication	Domain	0-3 months	6-12 months	>12 months	Group characteristics^a,b,c^	(Refs.)
Park *et al*, 2020; Wei *et al*, 2021; Lin *et al*, 2023	Physical performance and functional capacity	Decline	Improvement	Improvement, but no baseline levels	Park *et al*: Advanced stage (II-III); MIS; 65% received adjuvant therapy. Wei *et al*: Mixed stage (I-III); MIS. Lin *et al*: Mixed stage (I-III); MIS; no neoadjuvant therapy; adjuvant chemotherapy in 58% (62.5% TLTG, 56.3% LATG)	(9,26,28)
Tanaka *et al*, 2024; van der Wielen *et al*, 2022	Physical performance and functional capacity	Decline	Improvement	Baseline	Tanaka *et al*: Early stage (IA-IIB); MIS and open. Van der Wielen *et al*: Mixed stage (I-III); MIS and open; neoadjuvant therapy.	(17,29)
Tanaka *et al*, 2024; van der Wielen *et al*, 2022; Lin *et al*, 2023	Role engagement and daily living	Decline	Improvement, but no baseline	Stabilisation by year 3	Tanaka *et al*: Early stage (IA-IIB); MIS and open. Van der Wielen *et al*: Mixed stage (I-III); MIS and open; neoadjuvant therapy. Lin *et al*: Mixed stage (I-III); MIS; no neoadjuvant therapy; adjuvant chemotherapy in 58% (62.5% TLTG, 56.3% LATG).	(17,28,29)
Park *et al*, 2020	Role engagement and daily living	Decline	Decline	Decline	Advanced stage (II-III); MIS; 65% received adjuvant therapy.	(9)
Park *et al*, 2020	Social integration and interaction	Improvement	Peak at 12 months	Decline	Advanced stage (II-III); MIS; 65% received adjuvant therapy.	(9)
Van der Wielen *et al*, 2022	Social integration and interaction	Decline during first 6 months	OTG returned to baseline by 12 months; MITG remained below baseline	OTG exceeded baseline; MITG did not	Mixed stage (I-III); MIS and open; neoadjuvant therapy.	(29)
Tanaka *et al*, 2024	Emotional and psychological well-being	Decline	Improvement	N/A	Early stage (IA-IIB); MIS and open.	(17)
Yan *et al*, 2023; Lu *et al*, 2022; Lin *et al*, 2023	Emotional and psychological well-being	No significant change	No significant change	No significant change	Yan *et al*: Mixed stage (I-III); MIS. Lu *et al*: Mixed stage (I-III); MIS. Lin *et al*: Mixed stage (I-III); MIS; no neoadjuvant therapy; adjuvant chemotherapy in 58% (62.5% TLTG, 56.3% LATG).	(27,29,30)
Park *et al*, 2020	Emotional and psychological well-being	Improvement	Improvement	Slow decline after 1-2 years	Advanced stage (II-III); MIS; 65% received adjuvant therapy.	(9)
Park *et al*, 2020	Emotional and psychological well-being (body image)	Improvement	Peak at 1 year	Decline	Advanced stage (II-III); MIS; 65% received adjuvant therapy.	(9)
Van der Wielen *et al*, 2022	Emotional and psychological well-being (body image)	No significant change (OTG); transient improvement (MIS)	Gradual return to baseline (MIS)	Baseline	Mixed stage (I-III); MIS and open; neoadjuvant therapy.	(29)
Wei *et al*, 2021; Yan *et al*, 2023	Emotional and psychological well-being (body image)	No significant change	No significant change	No significant change	Mixed stage (I-III); MIS.	(26,27)
Park *et al*, 2020	Cognitive resilience	Decline during first year	Minimal improvement	Minimal improvement up to 3 years	Advanced stage (II-III); MIS; 65% received adjuvant therapy	(9)
Tanaka *et al*, 2024; Wei *et al*, 2021; Yan *et al*, 2023; Lu *et al*, 2022; van der Wielen *et al*, 2022	Cognitive resilience	No significant change	No significant change	No significant change	Tanaka *et al*: Early stage (IA-IIB); MIS and open. Wei *et al*: Mixed stage (I-III); MIS. Yan *et al*: Mixed stage (I-III); MIS. Lu *et al*: Mixed stage (I-III); MIS. Van der Wielen *et al*: Mixed stage (I-III); MIS and open; neoadjuvant therapy.	(17,26,27,29,30)
Van der Wielen *et al*, 2022	Global health perception and life satisfaction^d^	Decline	Improvement	Return to baseline only in OTG	Mixed stage (I-III); MIS and open; neoadjuvant therapy.	(29)
Park *et al*, 2020	Global health perception and life satisfactiond	Improvement	Improvement	Improvement	Advanced stage (II-III); MIS; 65% received adjuvant therapy.	(9)
Park *et al*, 2020	Economic consequences	No significant change	No significant change	Increase in year 2, recovery by year 3	Advanced stage (II-III); MIS; 65% received adjuvant therapy.	(9)
Wei *et al*, 2021; Yan *et al*, 2023; Lin *et al*, 2023; Tanaka *et al*, 2024; van der Wielen *et al*, 2022; Lu *et al*, 2022	Economic consequences	No significant change	No significant change	No significant change	Wei *et al*: Mixed stage (I-III); MIS. Yan *et al*: Mixed stage (I-III); MIS. Lin *et al*: Mixed stage (I-III); MIS; no neoadjuvant therapy; adjuvant chemotherapy in 58% (62.5% TLTG, 56.3% LATG). Tanaka *et al*: Early stage (IA-IIB); MIS and open. Van der Wielen *et al*: Mixed stage (I-III); MIS and open; neoadjuvant therapy. Lu *et al*: Mixed stage (I-III); MIS.	(17,26-30)

^a^Pre-operative data were available only in the studies by Park *et al* (9), Yan *et al* (27), Tanaka *et al* (17) and Van der Wielen *et al* (29).

^b^Chronological patterns could be directly extracted from Park *et al* (9) and Van der Wielen *et al* (29) in the remaining studies, only isolated post-operative time points were reported, and conclusions regarding trajectories are therefore indirect.

^c^Information on neoadjuvant and adjuvant therapy is included only where explicitly reported in the original studies, and other studies did not provide relevant data.

^d^No information was provided about the chronological course of dissatisfaction. LATG, laparoscopic-assisted total gastrectomy; LS, linear stapler; MIS, minimally invasive surgery; MITG, minimally invasive total gastrectomy; N/A, not available; OTG, open total gastrectomy; PP-RY, pouch Roux-en-Y; R-Y, Roux-en-Y; TLTG, totally laparoscopic total gastrectomy.

**Table IV:** Comparative findings in functional and single-item quality of life following gastrectomy.

Authors, year of publication	Domain	Comparison	Findings	Group characteristics^a^	(Refs.)
Wei *et al*, 2021; Yan *et al*, 2023	Physical performance and functional capacity	Stapling technique (LS vs. CS)	No significant differences	Wei *et al*: Mixed stage (I-III); MIS. Yan *et al*: Mixed stage (I-III); MIS	(26,27)
Tanaka *et al*, 2024; Van der Wielen *et al*, 2022; Lin *et al*, 2023	Physical performance and functional capacity	MIS vs. open	No significant differences	Tanaka *et al*: Early stage (IA-IIB); MIS and open. Van der Wielen *et al*: Mixed stage (I-III); MIS and open; neoadjuvant therapy. Lin *et al*: Mixed stage (I-III); MIS; no neoadjuvant therapy; adjuvant chemotherapy in 58% (62.5% TLTG, 56.3% LATG).	(17,28,29)
Kubota *et al*, 2024; Lu *et al*, 2022	Physical performance and functional capacity	Pouch vs. no pouch	No significant differences	Kubota *et al*: Mixed stage (I-III); MIS. Lu *et al*: Mixed stage (I-III); MIS.	(16,30)
Lin *et al*, 2023	Role engagement and daily living	TLTG vs. LATG	TLTG patients had better return-to-work ability at 6-12 months; no difference at 3 months	Mixed stage (I-III); MIS; no neoadjuvant therapy; adjuvant chemotherapy in 58% (62.5% TLTG, 56.3% LATG).	(28)
Van der Wielen *et al*, 2022	Role engagement and daily living	MIS vs. open	Open group showed persistently lower scores compared with MIS, but not statistically significant	Mixed stage (I-III); open and MIS; neoadjuvant therapy	(29)
Tanaka *et al*, 2024; Van der Wielen *et al*, 2022	Social integration and interaction	Surgical approach; MIS vs. open	Open group had higher scores thanlaparoscopic group; OTG back to baseline at 6 months, MITG declined from 3 months onwards	Early stage (IA-IIB); open and MIS, mixed stage (I-III); open and MIS; neoadjuvant therapy.	(17,29)
Lin *et al*, 2023	Social integration and interaction	Surgical approach; TLTG vs. LATG	TLTG associated with better scores throughout first 12 months, but no significant differences at assessed time points	Mixed stage (I-III); MIS; no neoadjuvant therapy; adjuvant chemotherapy in 58% (62.5% TLTG, 56.3% LATG).	(28)
Lu *et al*, 2022; Saeki *et al*, 2024; Kubota *et al*, 2024	Social integration and interaction	Body image; PP-RY vs. RY; CS vs. LS; aboral pouch (elderly vs. non-elderly)	No significant differences across comparisons	Lu *et al*: Mixed stage (I-III); MIS. Saeki *et al*: Early stage (IA-IIB); MIS and open. Kubota *et al*: Mixed stage (I-III); MIS.	(16,21,30)
Saeki *et al*, 2024	Social integration and interaction	Meal frequency	Increased need for meals may have affected daily living patterns	Early stage (IA-IIB); MIS and open.	(21)
Lu *et al*, 2022	Emotional and psychological well-being	PP-RY vs. RY	PP-RY associated with significantly improved anxiety scores compared with standard RY	Mixed stage (I-III); MIS.	(30)
Wei *et al*, 2021	Emotional and psychological well-being	Stapling technique (CS vs. LS)	CS patients more likely to experience anxiety at 12 months compared to LS, although not statistically significant; no significant differences in body image	Mixed stage (I-III); MIS.	(26)
Yan *et al*, 2023; van der Wielen *et al*, 2022; Lin *et al*, 2023	Emotional and psychological well-being	MIS vs. open	Some MIS cohorts reported slightly improved emotional scores at 6 months, but no significant differences overall; open group reported worse body image scores than laparoscopic, although not statistically significant	Yan *et al*: Mixed stage (I-III); MIS. Van der Wielen *et al*: Mixed stage (I-III); open and MIS; neoadjuvant therapy. Lin *et al*: Mixed stage (I-III); MIS; no neoadjuvant therapy; adjuvant chemotherapy in 58% (62.5% TLTG, 56.3% LATG).	(27-29)
Kubota *et al*, 2024; Lu *et al*, 2022	Emotional and psychological well-being	Pouch reconstructions	No significant differences in emotional or body image outcomes	Kubota *et al*: Mixed stage (I-III); MIS. Lu *et al*: Mixed stage (I-III); MIS.	(16,30)
Yan *et al*, 2023; Tanaka *et al*, 2024; Lin *et al*, 2023; Lu *et al*, 2022	Cognitive resilience	Stapling technique (CS vs. LS); MIS vs. open; Pouch vs. no pouch reconstruction	No significant differences	Yan *et al*: Mixed stage (I-III); MIS. Tanaka *et al*: Early stage (IA-IIB); open and MIS. Lin *et al*: Mixed stage (I-III); MIS; no neoadjuvant therapy; adjuvant chemotherapy in 58% (62.5% TLTG, 56.3% LATG). Lu *et al*: Mixed stage (I-III); MIS.	(17,27,28,30)
Lin *et al*, 2023	Global health perception and life satisfaction	TLTG vs. LATG	TLTG patients showed earlier improvements compared with LATG	Mixed stage (I-III); MIS; no neoadjuvant therapy; adjuvant chemotherapy in 58% (62.5% TLTG, 56.3% LATG).	(28)
Van der Wielen *et al*, 2022	Global health perception and life satisfaction	MIS vs. open	MIS associated with higher global health scores at 1 year; consistently better than open gastrectomy	Mixed stage (I-III); open and MIS; neoadjuvant therapy.	(29)
Lu *et al*, 2022	Global health perception and life satisfaction	PP-RY vs. RY	PP-RY reconstructions associated with better overall QoL compared with standard RY	Mixed stage (I-III); MIS.	(30)
Kubota *et al*, 2024; Tanaka *et al*, 2024; Saeki *et al*, 2024	Global health (dissatisfaction^b^)	Stapling technique (CS vs. LS); elderly vs. non-elderly; MIS vs. open	No significant differences in dissatisfaction scores across comparisons	Kubota *et al*: Mixed stage (I-III); MIS. Tanaka *et al*: Early stage (IA-IIB); open and MIS. Saeki *et al*: Early stage (IA-IIB); MIS and open.	(16,17,21)
Wei *et al*, 2021; an *et al*, 2023	Economic consequences	Stapling technique (LS vs. CS)	LS associated with better financial scores at 12 months compared with CS	Wei *et al*: Mixed stage (I-III); MIS. Yan *et al*: Mixed stage (I-III); MIS.	(26,27)
Tanaka *et al*, 2024	Economic consequences	MIS vs. open	Open group reported higher burden scores, laparoscopic lower scores; differences not statistically significant	Early stage (IA-IIB); MIS and open.	(17)

^a^Information on adjuvant and neoadjuvant therapy is included in the table when reported in the original studies.

^b^For the studies using the PGSAS questionnaire. CS, circular stapler; HRM, high-resolution manometry; LATG, laparoscopic-assisted total gastrectomy; LS, linear stapler; MIS, minimally invasive surgery; MITG, minimally invasive total gastrectomy; OTG, open total gastrectomy; PP-RY, proximal jejunal pouch Roux-en-Y; PGSAS, postgastrectomy syndrome assessment scale; QoL, quality of life; RY, Roux-en-Y; TLTG, totally laparoscopic total gastrectomy.

**Table V:** Chronological patterns of symptom-related quality of life following gastrectomy.

Authors, year of publication	Symptom	0-6 months	6-12 months	>12 months	Group characteristics^a,b,c^	(Refs).
Park *et al*, 2020	Fatigue	Peak	Plateau	Return to baseline by year 3	Advanced stage (II-III); MIS; 65% received adjuvant therapy	(9)
Van der Wielen *et al*, 2022	Fatigue	Peak	Gradual return to baseline	Baseline	Mixed stage (I-III); MIS and open; neoadjuvant therapy	(29)
Van der Wielen *et al*, 2022	Nausea/vomiting	Peak	Return near baseline by 6 months	Stable by year 3	Mixed stage (I-III); MIS and open; neoadjuvant therapy	(29)
Park *et al*, 2020	Nausea/vomiting	No significant change	No significant change	No significant change	Advanced stage (II-III); MIS; 65% received adjuvant therapy	(9)
Van der Wielen *et al*, 2022	Dyspnoea	Peak	Return near baseline by 6 months	Stable by year 3	Mixed stage (I-III); MIS and open; neoadjuvant therapy	(29)
Park *et al*, 2020	Dyspnoea	No significant change	No significant change	No significant change	Advanced stage (II-III); MIS; 65% received adjuvant therapy	(9)
Park *et al*, 2020	Pain	Peak	Improvement	Secondary peak in year 2, slight improvement after secondary peak	Advanced stage (II-III); MIS; 65% received adjuvant therapy	(9)
Tanaka *et al*, 2024	Pain	Peak	Return to baseline by 12 months	Baseline	Early stage (IA-IIB); MIS and open	(17)
Van der Wielen *et al*, 2022	Pain	Peak	Return to baseline by 12 months for the open group, MIS remained elevated	Baseline for open surgery, elevated for MIS	Mixed stage (I-III); MIS and open; neoadjuvant therapy	(29)
Wei *et al*, 2021; Yan *et al*, 2023	Pain	Not reported	Improved scores at 6-12m	Not reported	Mixed stage (I-III); MIS; proportion receiving neoadjuvant therapy not reported.	(26,27)
Park *et al*, 2020; van der Wielen *et al*, 2022	Constipation	No change	No change	No change	Park *et al*: Advanced stage (II-III); MIS; 65% received adjuvant therapy. Van der Wielen *et al*: Mixed stage (I-III); MIS and open; neoadjuvant therapy.	(9,29)
Wei *et al*, 2021; Yan *et al*, 2023; van der Wielen *et al*, 2022	Diarrhoea	Decline; worse in neoadjuvant patients at 3 months	Return to base line by 6–12 months	Baseline	Wei *et al*: Mixed stage (I-III); MIS. Yan *et al*: Mixed stage (I-III); MIS; proportion receiving neoadjuvant therapy not reported. Van der Wielen *et al*: Mixed stage (I-III); MIS and open; neoadjuvant therapy.	(26,27,29)
Park *et al*, 2020	Diarrhoea	Decline; worse in neoadjuvant patients at 3 months	Elevated	Elevated up to 2 years	Advanced stage (II-III); MIS; 65% received adjuvant therapy.	(9)
Park *et al*, 2020; van der Wielen *et al*, 2022	Dysphagia	Peak immediately postoperatively and at 3 months	Return to baseline by 6 months	No change	Park *et al*: Advanced stage (II-III); MIS; 65% received adjuvant therapy. Van der Wielen *et al*: Mixed stage (I-III); MIS and open; neoadjuvant therapy.	(9,29)
Park *et al*, 2020; van der Wielen *et al*, 2022; Lin *et al*, 2023	Reflux	Increased	Remained elevated; improvement noted TLTG vs. LATG	Remained elevated	Park *et al*: Advanced stage (II-III); MIS; 65% received adjuvant therapy. Van der Wielen *et al*: Mixed stage (I-III); MIS and open; neoadjuvant therapy. Lin *et al*: Mixed stage (I-III); MIS; no neoadjuvant therapy; adjuvant chemotherapy in 58% (62.5% TLTG, 56.3% LATG).	(9,28,29)
Park *et al*, 2020; van der Wielen *et al*, 2022	Eating restrictions	Decline	No improvement	Persisted in MITG; improved in OTG	Park *et al*: Advanced stage (II-III); MIS; 65% received adjuvant therapy. Van der Wielen *et al*: Stages I-III; MIS and open; neoadjuvant therapy.	(9,29)
Park *et al*, 2020; van der Wielen *et al*, 2022; Wei *et al*, 2021; Yan *et al*, 2023; Lin *et al*, 2023	Taste disturbances	Peak; worse in MIS	Peak; more evident in neoadjuvant patients at 6 months	No significant further changes	Park *et al*: Advanced stage (II-III); MIS; 65% received adjuvant therapy. Van der Wielen *et al*: Mixed stage (I-III); MIS and open; neoadjuvant therapy. Wei *et al*: Mixed stage (I-III); MIS. Yan *et al*: Mixed stage (I-III); MIS; proportion receiving neoadjuvant therapy not\ reported. Lin *et al*: Mixed stage (I-III); MIS; no neoadjuvant therapy; adjuvant chemotherapy in 58% (62.5% TLTG, 56.3% LATG).	(9,26-29)
Park *et al*, 2020; van der Wielen *et al*, 2022; Lin *et al*, 2023	Appetite loss	No significant change; worse scores in neoadjuvant patients	Recovery to baseline by 6 months; better scores after PP-RY	No change	Park *et al*: Advanced stage (II-III); MIS; 65% received adjuvant therapy. Van der Wielen *et al*: Stages I-III; MIS and open; neoadjuvant therapy. Lin *et al*: Mixed stage (I-III); MIS; no neoadjuvant therapy; adjuvant chemotherapy in 58% (62.5% TLTG, 56.3% LATG).	(9,28,29)
Park *et al*, 2020; van der Wielen *et al*, 2022	Sleep	Stable; worse in MIS	Recovery to baseline by 6 months	Baseline	Park *et al*: Advanced stage (II-III); MIS; 65% received adjuvant therapy. Van der Wielen *et al*: Mixed stage (I-III); MIS and open; neoadjuvant therapy.	(9,29)
Park *et al*, 2020	Mouth dryness	Increased; worse in MIS	Increased	Slight improvement after 2 years	Advanced stage (II-III); MIS; 65% received adjuvant therapy.	(9)
Van der Wielen *et al*, 2022	Mouth dryness	Increased; worsening scores in MIS for the first 3 months	Recovery to baseline by 6 months	Baseline	Mixed stage (I-III); MIS and open; neoadjuvant therapy.	(29)
Park *et al*, 2020	Hair loss	Worsening	Worsening	Improved after year 2	Advanced stage (II-III); MIS; 65% received adjuvant therapy.	(9)
Tanaka *et al*, 2024	Dumping	No significant findings	No significant findings	No significant findings	Early stage (IA-IIB); MIS and open.	(17)

^a^Pre-operative data were available only in the studies by Park *et al* (9), Yan *et al* (26), Tanaka *et al* (17) and Van der Wielen *et al* (28).

^b^Chronological patterns could be directly extracted only from Park *et al* (9) and Van der Wielen *et al* (28); in the remaining studies, only isolated postoperative time points were reported, and conclusions regarding trajectories are therefore indirect.

^c^Information on neoadjuvant and adjuvant therapy is included only where explicitly reported in the original studies; other studies did not provide relevant data. CS, circular stapler; DG, distal gastrectomy; HRM, high-resolution manometry; LATG, laparoscopic-assisted total gastrectomy; LS, linear stapler; MIS, minimally invasive surgery; PP-RY, Pouch Roux-en-Y reconstruction; QoL, quality of life; RY, Roux-en-Y reconstruction; TG, total gastrectomy; TLTG, totally laparoscopic total gastrectomy.

**Table VI:** Comparative findings in symptom-associated quality of life following gastrectomy.

			Statistically significant findings of symptom burden on surgical approach comparison														
Authors, year of publication	Group characteristics^a^	Comparison parameters	Fatigue	Nausea/vomiting, dyspnoea	Pain	Constipation	Diarrhoea	Dysphagia	Reflux	Eating restrictions, meal quantity	Taste disturbances	Appetite loss	Sleep disturbances	Mouth dryness	Hair loss	Dumping symptoms	(Refs.)
Wei *et a*l, 2021	Stages I-III, MIS	Linear stapler vs. circular stapler	No	No	No	Yes^e^	No	Yes^g^	No	No	No	No	No	No	No	No	(26)
Yan *et al*, 2023	Stages I-III, MIS	Linear stapler i) vs. circular stapler ii) Intracorporeal vs. extracorporeal anastomosis iii) Overlap vs. π-shaped vs. OrVil anastomosis	No	No	No	No	Yes^f^	Yes^g^	Yes^i^	Yes^j^	No	No	No	No	No	No	(27)
Lin *et al*, 2023	Stages I-III, MIS; no neoadjuvant therapy; adjuvant chemotherapy in 58% (62.5% TLTG, 56.3% LATG).	TLTG vs. LATG	No	No	No	No	No	Yes^h^	No	Yes^k^	No	No	No	No	No	No	(28)
Tanaka *et al*, 2024	Stages IA-IIB, open and MIS	Laparoscopic vs. open gastrectomy	Yes^b^	No	No	No	No	No	No	No	No	No	No	No	No	No	(17)
Van der Wielen *et al*, 2022	Stages I-III, neoadjuvant treatment	Open vs. MIS gastrectomy in neoadjuvant patients	No	No	No	No	No	No	No	No	No	No	No^n^	No	No	No	(29)
Saeki *et al*, 2024	Stages IA-IIB, open and MIS	i) HRM findings ii) Linear stapler vs. Circular Stapler	No	No	Yes^c^	No	No	No	No	No	No	No	No	No	No	No	(21)
Lu *et al*, 2022	Stages I-III, MIS	R-Y vs. PP-RY	No	No	Yes^d^	No	No	No	No	Yesl	No	Yes^m^	No	No	No	No	(30)
Kubota *et al*, 2024	Stages I-III, MIS	Aboral pouch in the elderly vs. non-elderly population	No	No	No	No	No	No	No	No	No	No	No	No	No	No	(16)
Park *et al*, 2020	Stages II-III, MIS, 65% adjuvant	TG vs. DG (TG data used only)	No	No	No	No	No	No	No	No	No	No	No	No	No	No	(9)

^a^Information on adjuvant and neoadjuvant therapy is included in the table when reported in the original studies.

^b^Marginal difference between laparoscopic and open approaches at 1 month; no difference at 12 months.

^c^Distal latency on HRM correlated with abdominal pain; no difference between CS and LS.

^d^PP-RY associated with lower chest and abdominal pain scores at 12 months.

^e^CS associated with worse scores than LS at 12 months.

^f^LS linked to higher incidence of diarrhoea at 3 months (MIS cohort).

^g^CS associated with higher dysphagia scores than LS at 12 months (MIS).

^h^TLTG associated with improved outcomes compared with LATG at 6 months.

^i^LS associated with worse reflux scores than CS at 12 months.

^j^More severe eating restrictions in CS compared with LS at 12 months (MIS).

^k^PP-RY associated with significantly improved scores compared with standard R-Y.

^l^Meal quantity greater in TLTG group from 3 months onward (advanced cancer, no neoadjuvant).

^m^PP-RY associated with improved appetite outcomes at 12 months compared with standard reconstruction.

^n^MITG associated with worse sleep scores, with recovery to baseline by 6 months. CS, circular stapler; DG, distal gastrectomy; HRM, high-resolution manometry; LATG, laparoscopic-assisted total gastrectomy; LS, linear stapler; MIS, minimally invasive surgery; MITG, minimally invasive total gastrectomy; PP-RY, Pouch Roux-en-Y reconstruction; RY, Roux-en-Y reconstruction; TG, total gastrectomy; TLTG, totally laparoscopic total gastrectomy.

## Data Availability

The data generated in the present study may be requested from the corresponding author.
